# Supplementary material for: Diversification and extinction of Hemiptera in deep time
Source: Commun Biol. 2025 Mar 3;8:352. doi: 10.1038/s42003-025-07773-x (PMC11876582; doi:10.1038/s42003-025-07773-x)
Supplement: Supplementary file 1 — Supplementary Information [file 42003_2025_7773_MOESM1_ESM.pdf]

Supplementary Information for

Diversification and extinction of Hemiptera in deep time

Mathieu Boderau, André Nel and Corentin Jouault

Corresponding author: Mathieu Boderau

Email: mathieuboderau@gmail.com

This PDF file includes:

Supplementary Figures 1 to 13
Supplementary Tables 1 to 8

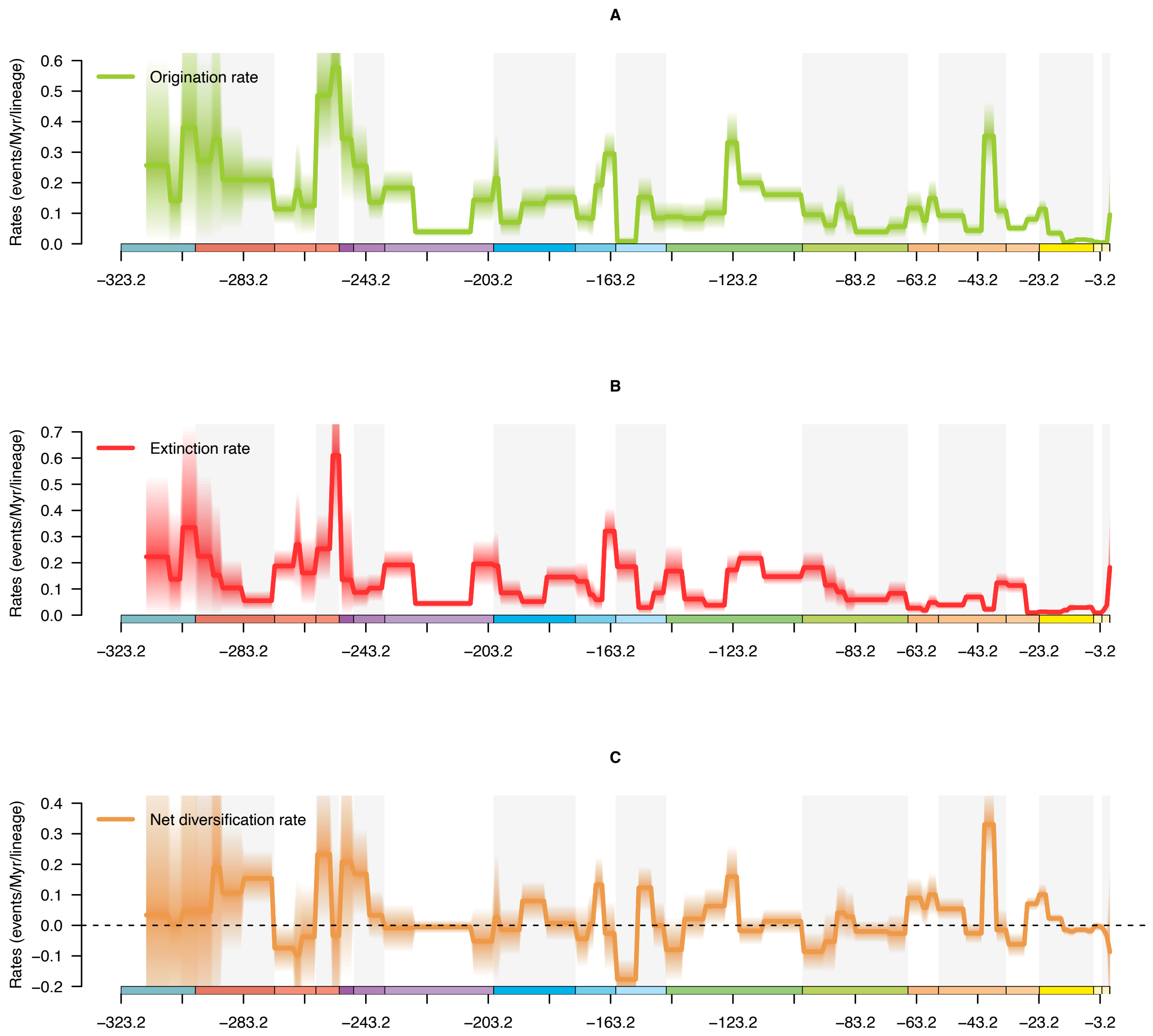

Supplementary Figure 1. Diversification and diversity dynamics of Hemiptera (genus-level analysis). **A**. Bayesian estimates of origination rates. **B**. Bayesian estimates of extinction rates. **C**. Net diversification rate. . BDCS analyses performed under geological stages time-bins. Solid lines indicate means posterior rates while shaded areas shows 95% CI. Time-unit is millions of years (Myr). Colors of geological periods in chronostratigraphic scale following International Chronostratigraphic Chart (ICS, v2023/09).

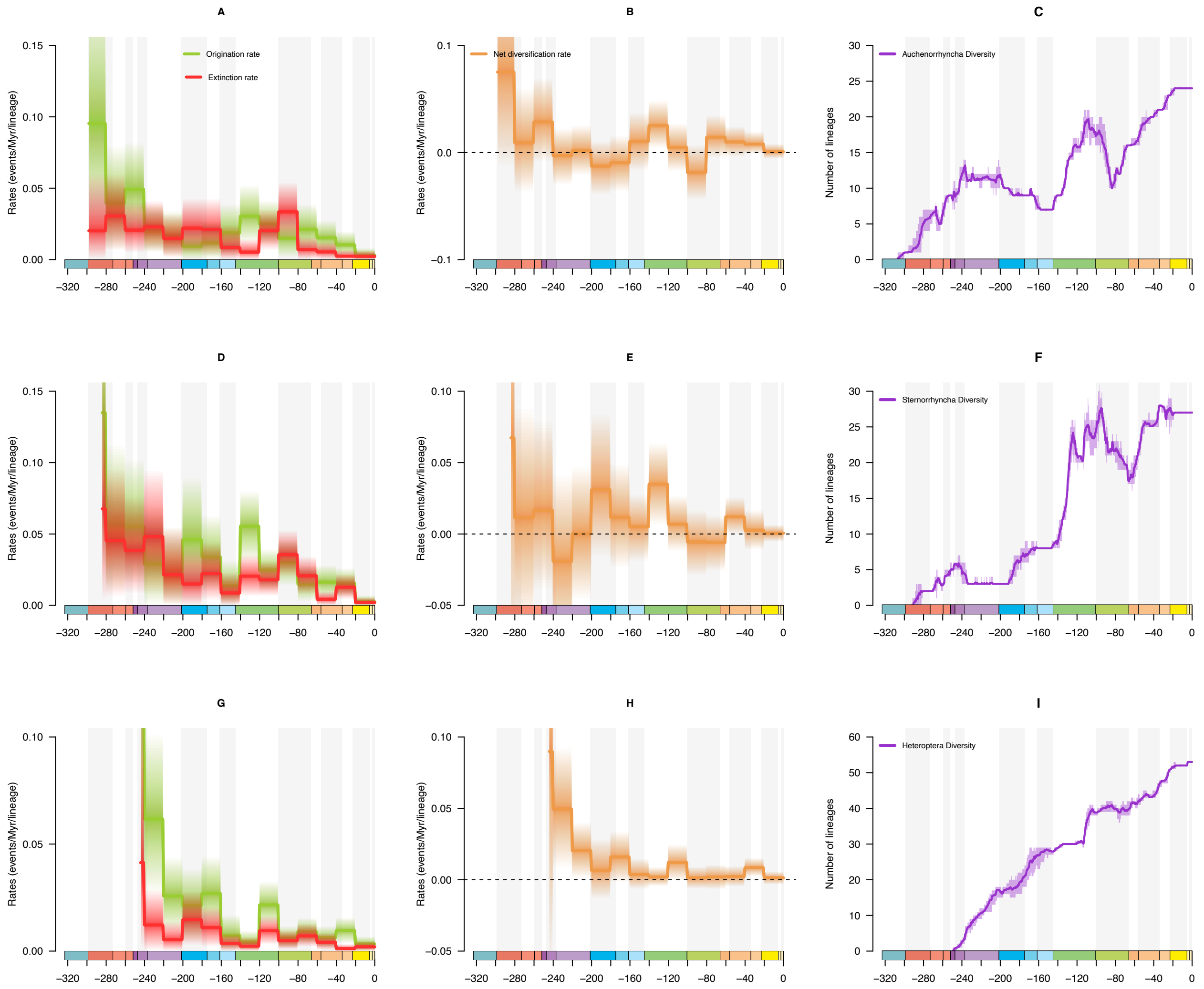

Supplementary Figure 2. Asynchronous diversification and diversity dynamics within Hemiptera (family-level analysis). **A.**, **D.**, **G.** Bayesian estimates of origination (green) and extinction (red) rates for Hemiptera major clades. **B.**, **E.**, **H.** Net diversification rate (origination minus extinction rates) for Hemiptera major clades. **C.**, **F.**, **I.** Diversity through time (number of genera) of Hemiptera major clades. Solid lines indicate means posterior rates while shaded areas shows 95% CI. Time-unit is millions of years (Ma). Colors of geological periods in chronostratigraphic scale following International Chronostratigraphic Chart (ICS, v2023/09).

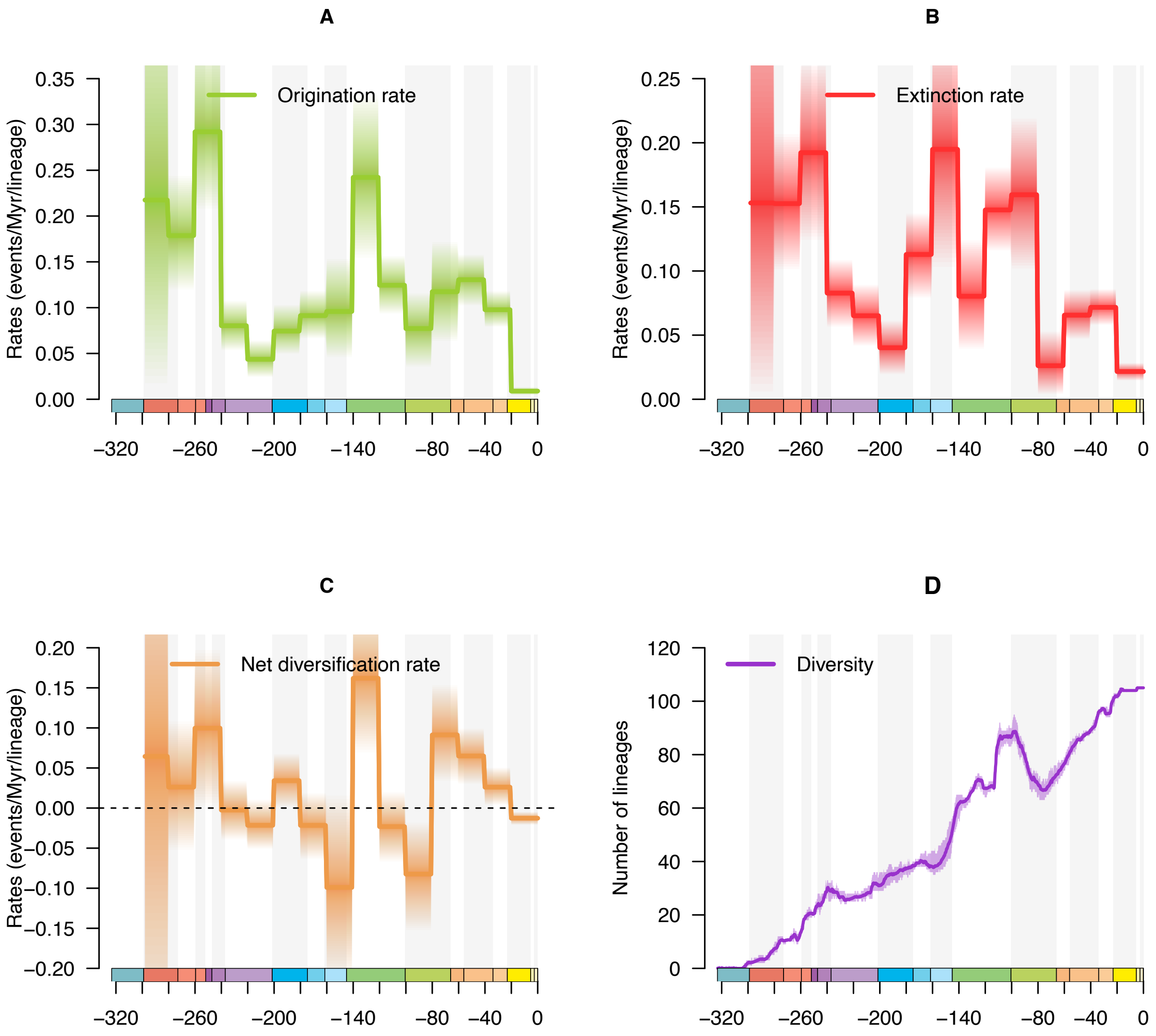

Supplementary Figure 3. Diversification and diversity dynamics within Hemiptera (family-level analysis). **A**. Bayesian estimates of origination rates. **B**. Bayesian estimates of extinction rates. **C**. Net diversification rate (origination minus extinction) rates for Hemiptera. **D**. Diversity through time (number of genera) of Hemiptera. Solid lines indicate means posterior rates while shaded areas shows 95% CI. ATR: Angiosperm Terrestrial Revolution. K-Pg: Cretaceous-Paleogene Event. Ol/Mi: Oligocene Miocene global cooling and drying. P/T: Permian-Triassic Event. T/J: Triassic-Jurassic Event. Time-unit is millions of years (Myr). Colors of geological periods in chronostratigraphic scale following International Chronostratigraphic Chart (ICS, v2023/09).

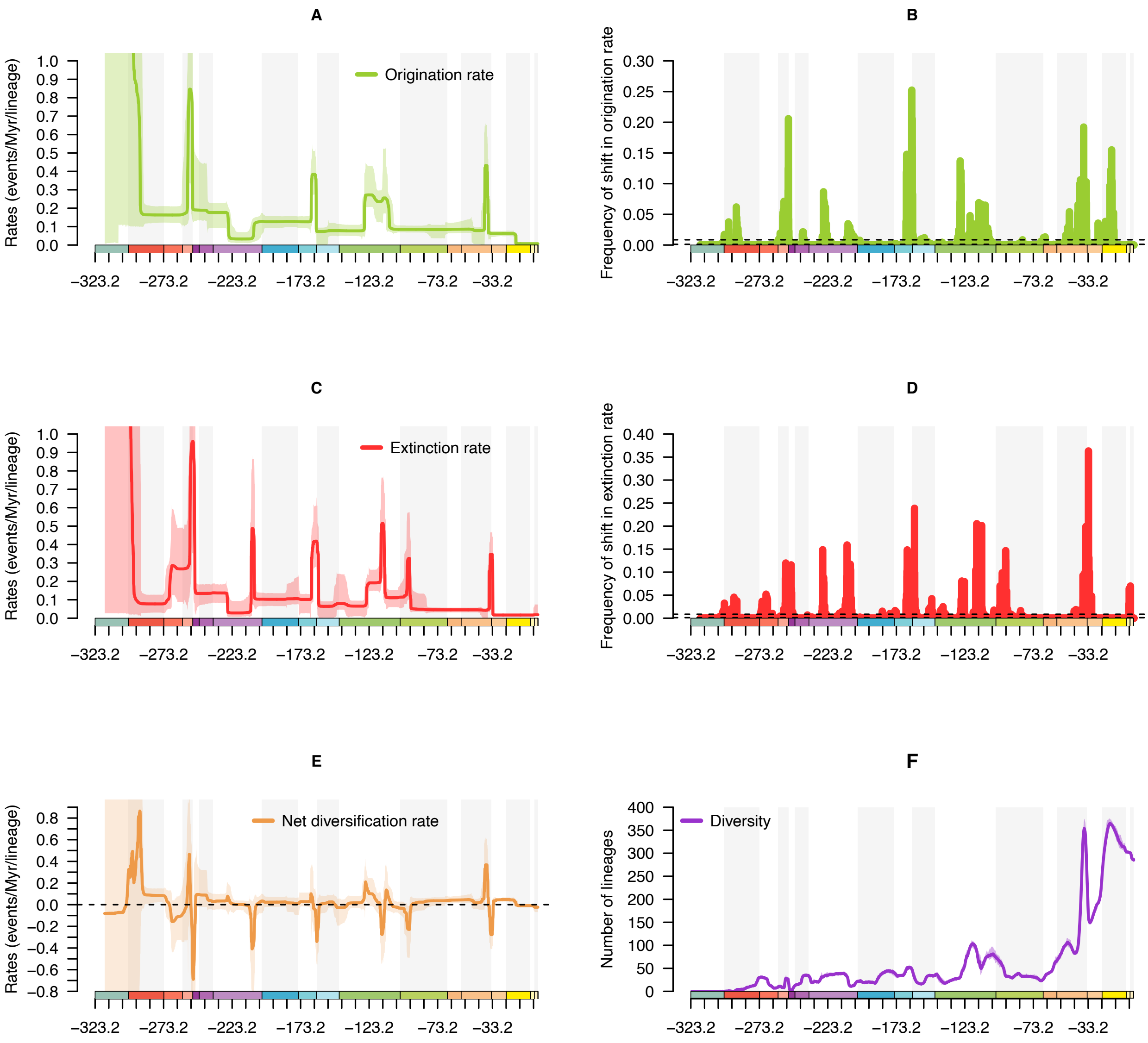

Supplementary Figure 4. Diversification and diversity dynamics of Hemiptera (genus-level analysis). Bayesian estimations of origination and extinction rates through time as inferred by PyRate using reversible jump Markov Chain Monte Carlo. Marginal estimates of origination rates (A) and extinction rates (C) through time shown as mean and 95% CI (shaded areas). Frequency of a sampled rate shift computed within small time bins for origination (B) and extinction rates (D), with horizontal dashed lines indicating log-Bayes factors of 2 (bottom) and 6 (top). Sampling frequencies higher than log-Bayes factors = 6 indicate strong statistical support for a rate shift. Panel (E) indicates posterior difference between origination and extinction rates through time defined as net diversification rates through time. Panel (F) represents number of genera through

time computed by summing up lifespans of all genera. Time-unit is millions of years (Myr). Colors of geological periods in chronostratigraphic scale following International Chronostratigraphic Chart (ICS, v2023/09).

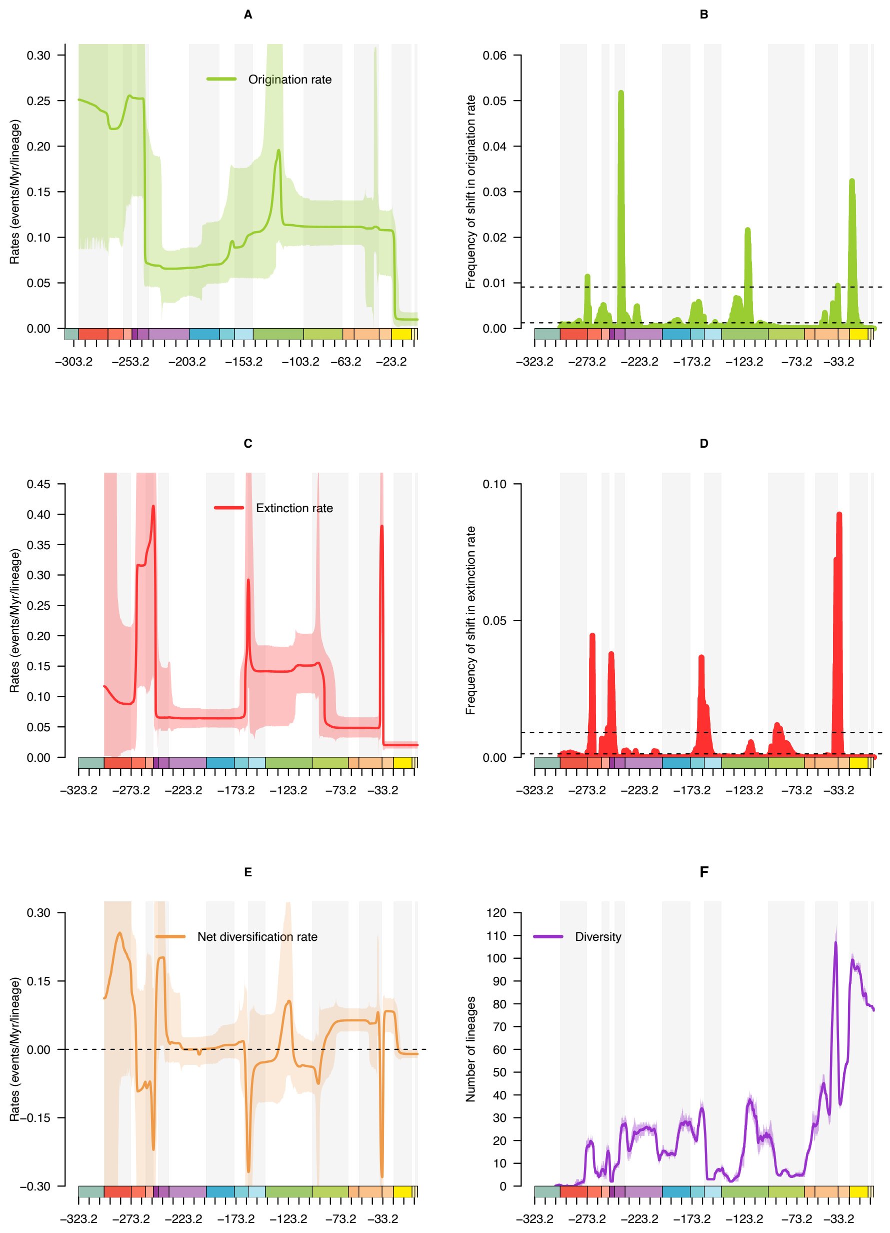

Supplementary Figure 5. Diversification and diversity dynamics of Auchenorrhyncha (genus-level analysis). Bayesian estimations of origination and extinction rates through time as inferred by PyRate using reversible jump Markov Chain Monte Carlo. Marginal estimates of origination rates (A) and extinction rates (C) through time shown as mean and 95% CI (shaded areas). Frequency of a sampled rate shift computed within small time bins for origination (B) and extinction rates (D), with horizontal dashed lines indicating log-Bayes factors of 2 (bottom) and 6 (top). Sampling frequencies higher than log-Bayes factors = 6 indicate strong statistical support for a rate shift. Panel (E) indicates posterior difference between origination and extinction rates through time

defined as net diversification rates through time. Panel (F) represents number of genera through time computed by summing up lifespans of all genera. Time-unit is millions of years (Myr). Colors of geological periods in chronostratigraphic scale following International Chronostratigraphic Chart (ICS, v2023/09).

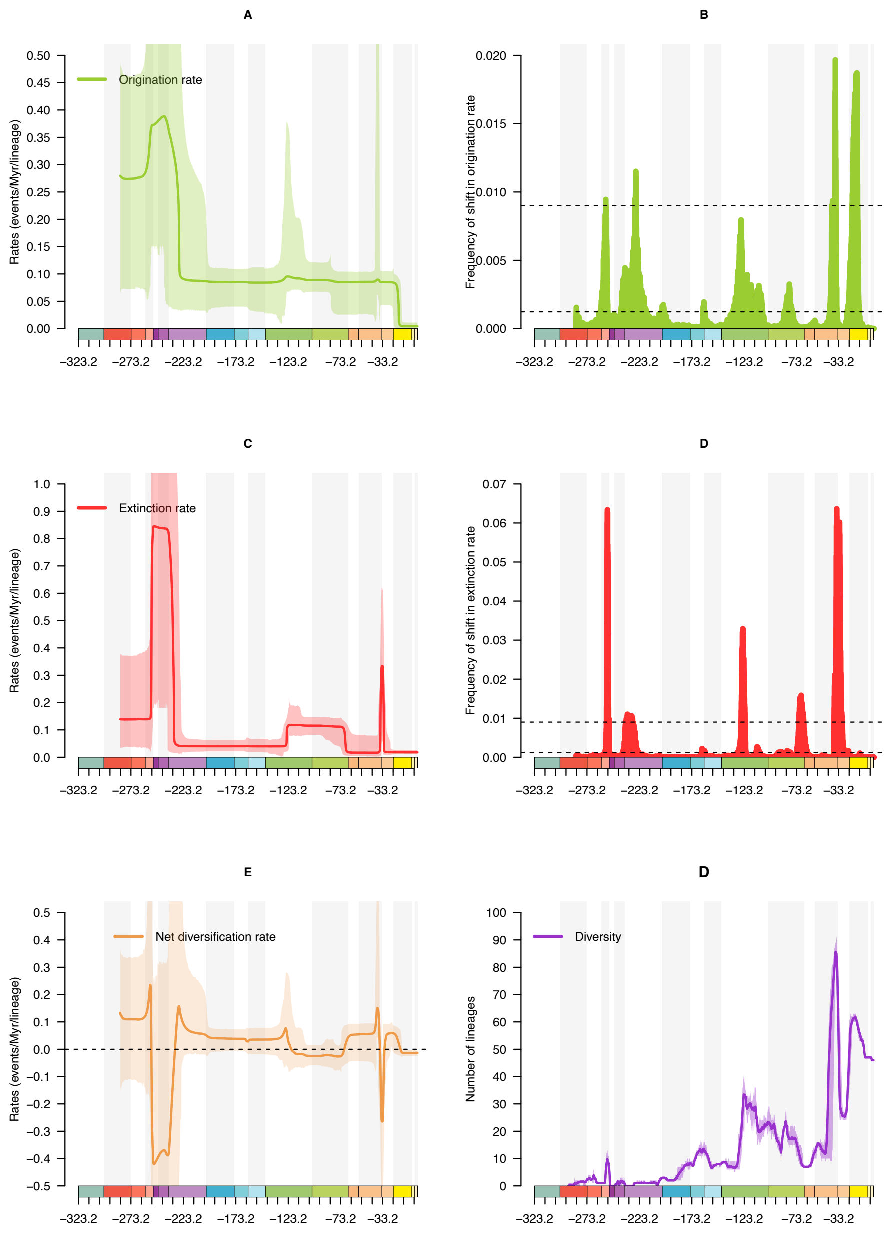

Supplementary Figure 6. Diversification and diversity dynamics of Sternorrhyncha (genus-level analysis). Bayesian estimations of origination and extinction rates through time as inferred by PyRate using reversible jump Markov Chain Monte Carlo. Marginal estimates of origination rates (A) and extinction rates (C) through time shown as mean and 95% CI (shaded areas). Frequency of a sampled rate shift computed within small time bins for origination (B) and extinction rates (D), with horizontal dashed lines indicating log-Bayes factors of 2 (bottom) and 6 (top). Sampling frequencies higher than log-Bayes factors = 6 indicate strong statistical support for a rate shift. Panel (E) indicates posterior difference between origination and extinction rates through time

defined as net diversification rates through time. Panel (F) represents number of genera through time computed by summing up lifespans of all genera. Time-unit is millions of years (Myr). Colors of geological periods in chronostratigraphic scale following International Chronostratigraphic Chart (ICS, v2023/09).

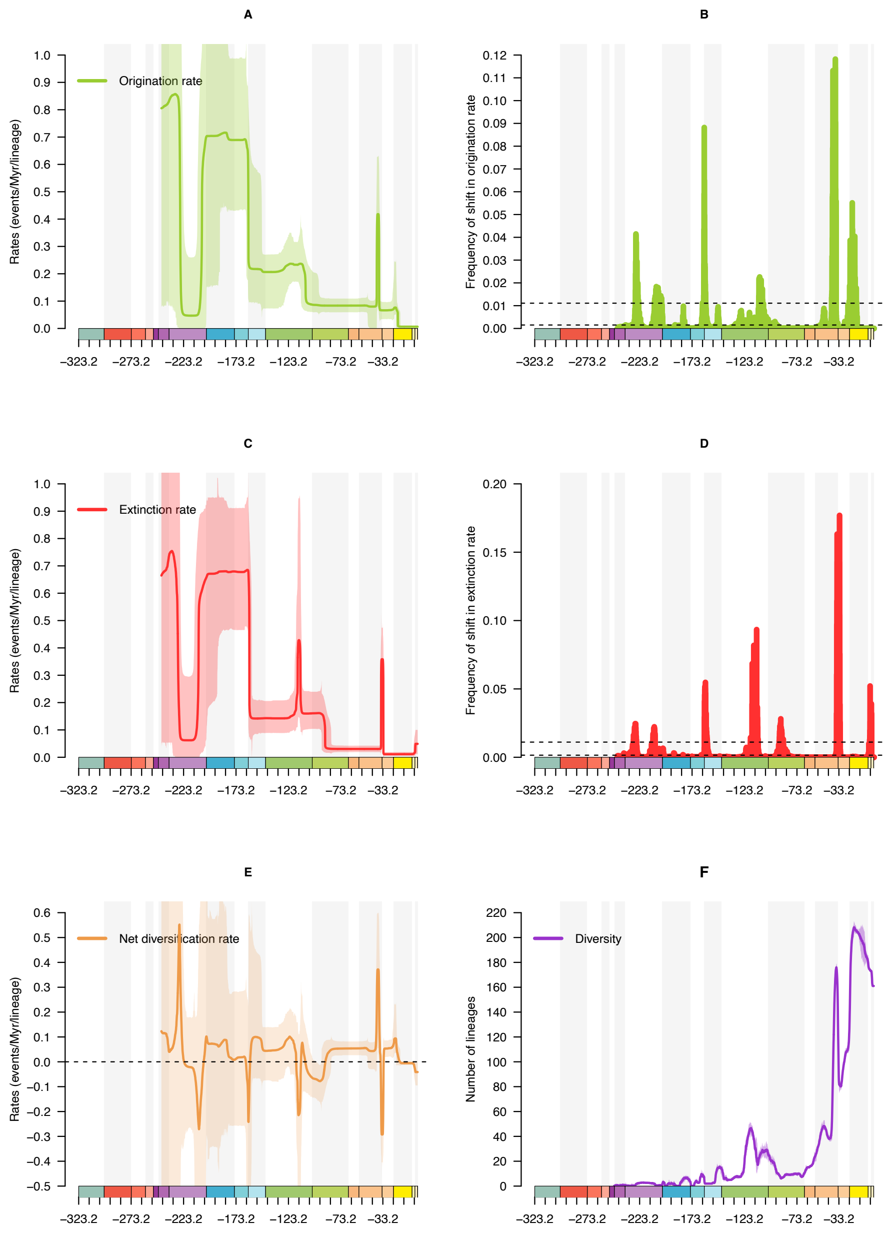

Supplementary Figure 7. Diversification and diversity dynamics of Heteroptera (genus-level analysis). Bayesian estimations of origination and extinction rates through time as inferred by PyRate using reversible jump Markov Chain Monte Carlo. Marginal estimates of origination rates (A) and extinction rates (C) through time shown as mean and 95% CI (shaded areas). Frequency of a sampled rate shift computed within small time bins for origination (B) and extinction rates (D), with horizontal dashed lines indicating log-Bayes factors of 2 (bottom) and 6 (top). Sampling frequencies higher than log-Bayes factors = 6 indicate strong statistical support for a rate shift. Panel (E) indicates posterior difference between origination and extinction rates through time

defined as net diversification rates through time. Panel (F) represents number of genera through time computed by summing up lifespans of all genera. Time-unit is millions of years (Myr). Colors of geological periods in chronostratigraphic scale following International Chronostratigraphic Chart (ICS, v2023/09).

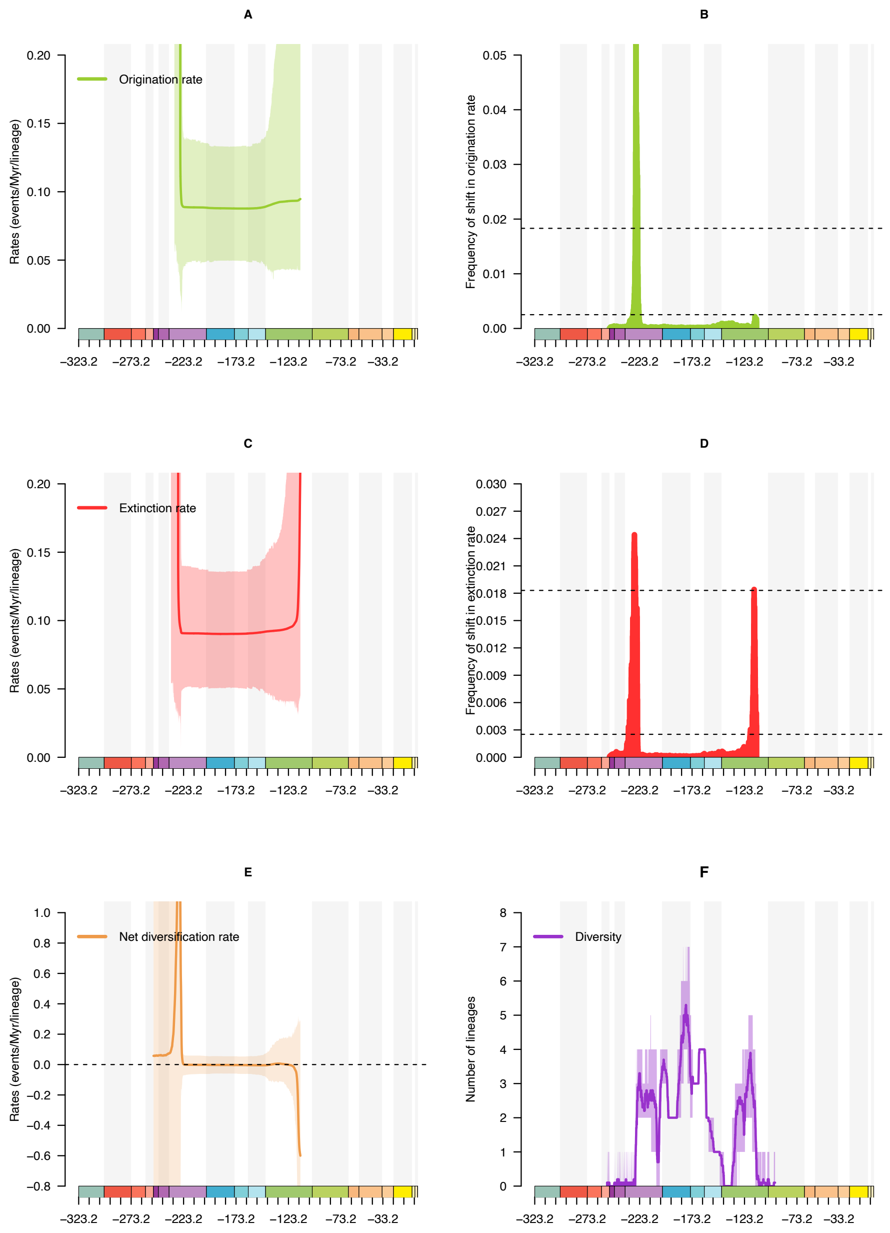

Supplementary Figure 8. Diversification and diversity dynamics of Coleorrhyncha (genus-level analysis). Bayesian estimations of origination and extinction rates through time as inferred by PyRate using reversible jump Markov Chain Monte Carlo. Marginal estimates of origination rates (A) and extinction rates (C) through time shown as mean and 95% CI (shaded areas). Frequency of a sampled rate shift computed within small time bins for origination (B) and extinction rates (D), with horizontal dashed lines indicating log-Bayes factors of 2 (bottom) and 6 (top). Sampling frequencies higher than log-Bayes factors = 6 indicate strong statistical support for a rate shift.

Panel (E) indicates posterior difference between origination and extinction rates through time defined as net diversification rates through time. Panel (F) represents number of genera through time computed by summing up lifespans of all genera. Time-unit is millions of years (Myr). Colors of geological periods in chronostratigraphic scale following International Chronostratigraphic Chart (ICS, v2023/09).

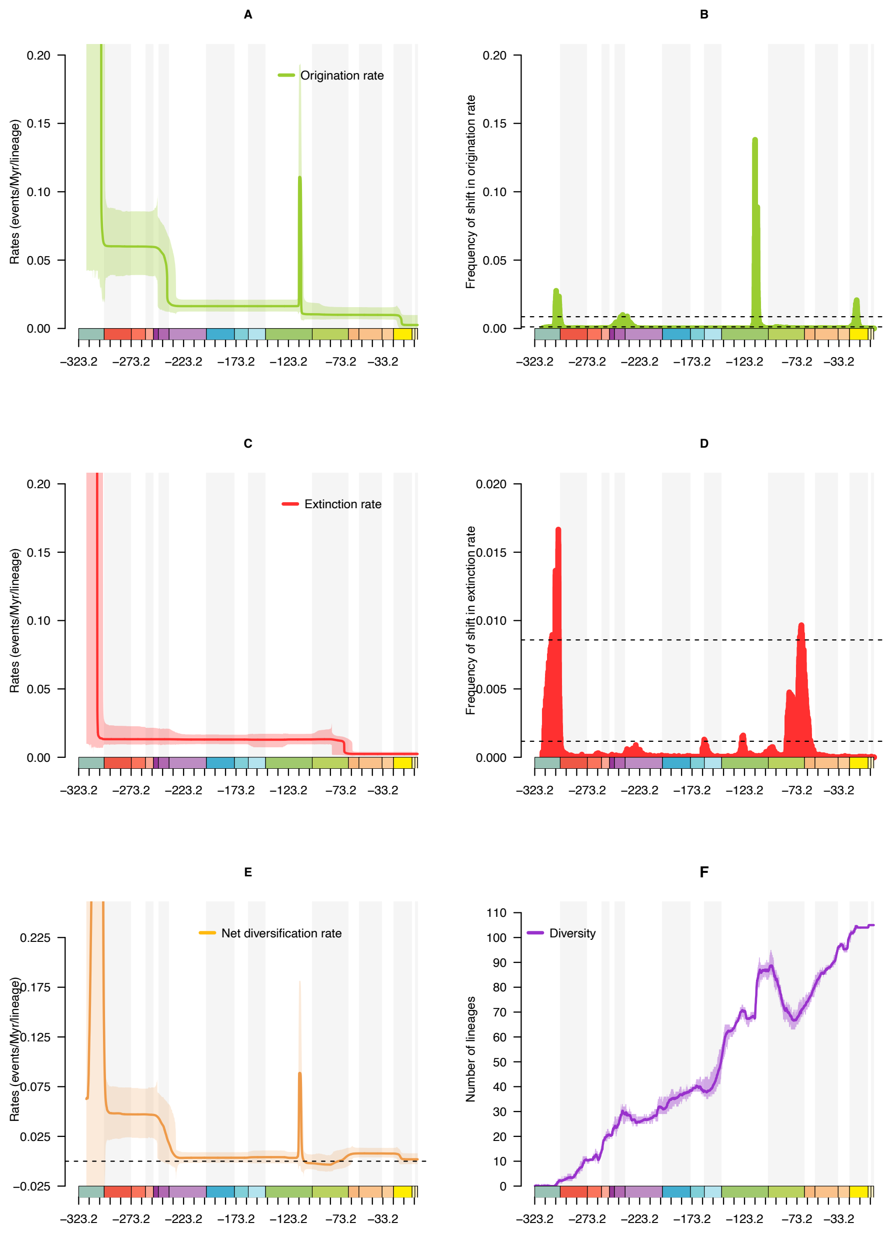

Supplementary Figure 9. Diversification and diversity dynamics of Hemiptera (family-level analysis). Bayesian estimations of origination and extinction rates through time as inferred by PyRate using reversible jump Markov Chain Monte Carlo. Marginal estimates of origination rates (A) and extinction rates (C) through time shown as mean and 95% CI (shaded areas). Frequency of a sampled rate shift computed within small time bins for origination (B) and extinction rates (D), with horizontal dashed lines indicating log-Bayes factors of 2 (bottom) and 6 (top). Sampling frequencies higher than log-Bayes factors = 6 indicate strong statistical support for a rate shift.

Panel (E) indicates posterior difference between origination and extinction rates through time defined as net diversification rates through time. Panel (F) represents number of families through time computed by summing up lifespans of all families. Time-unit is millions of years (Myr). Colors of geological periods in chronostratigraphic scale following International Chronostratigraphic Chart (ICS, v2023/09).

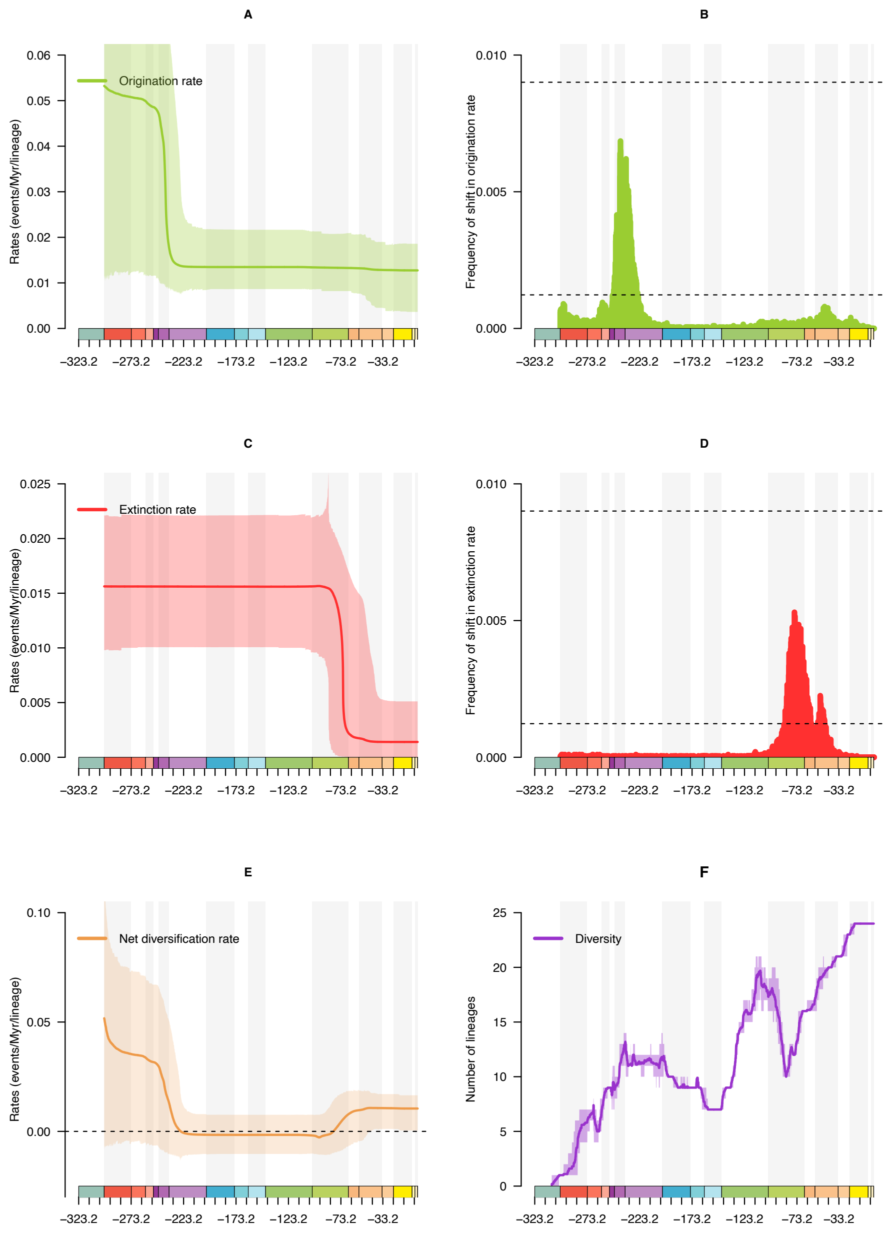

Supplementary Figure 10. Diversification and diversity dynamics of Auchenorrhyncha (family-level analysis). Bayesian estimations of origination and extinction rates through time as inferred by PyRate using reversible jump Markov Chain Monte Carlo. Marginal estimates of origination rates (A) and extinction rates (C) through time shown as mean and 95% CI (shaded areas). Frequency of a sampled rate shift computed within small time bins for origination (B) and extinction rates (D), with horizontal dashed lines indicating log-Bayes factors of 2 (bottom) and 6 (top). Sampling frequencies higher than log-Bayes factors = 6 indicate strong statistical support for a rate shift. Panel (E) indicates posterior difference between origination and extinction rates through time

defined as net diversification rates through time. Panel (F) represents number of genera through time computed by summing up lifespans of all genera. Time-unit is millions of years (Myr). Colors of geological periods in chronostratigraphic scale following International Chronostratigraphic Chart (ICS, v2023/09).

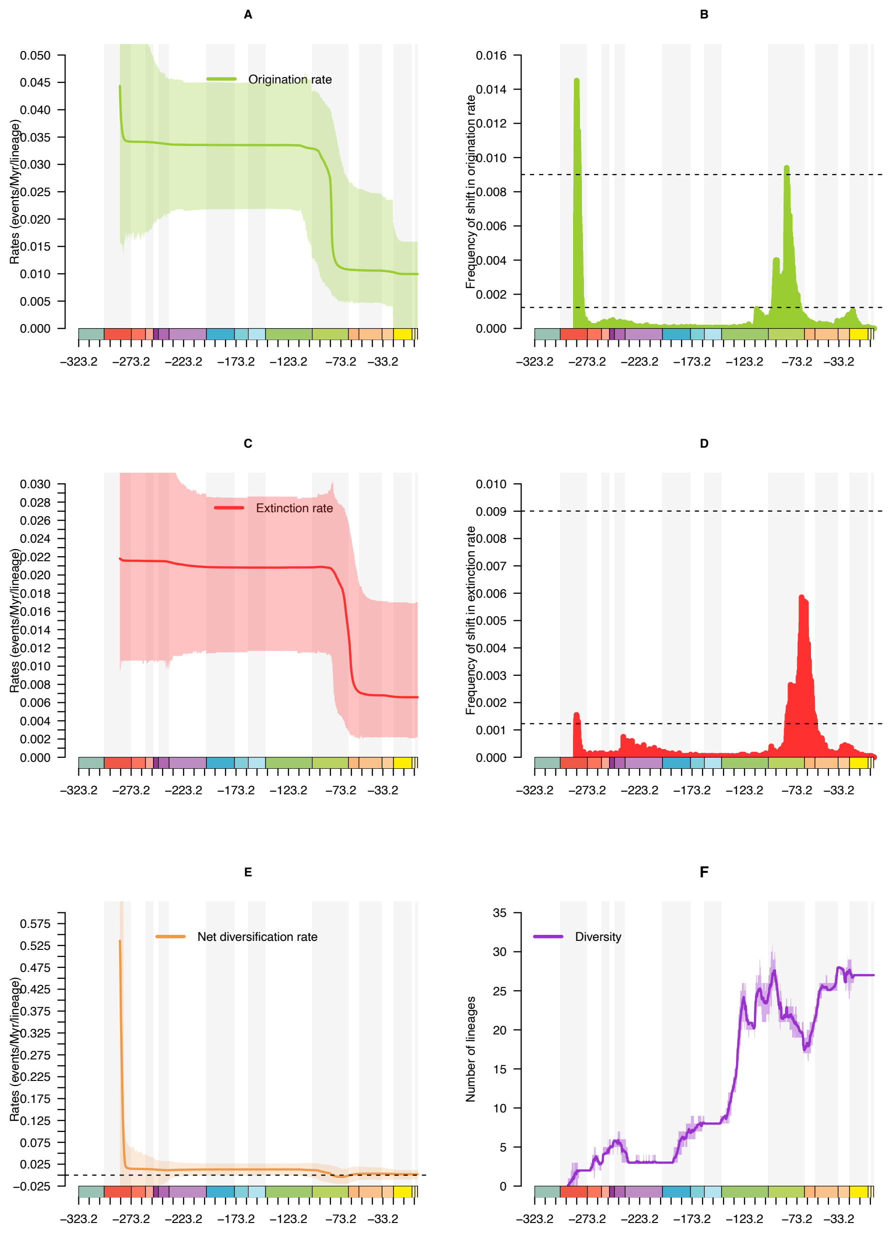

Supplementary Figure 11. Diversification and diversity dynamics of Sternorrhyncha (family-level analysis). Bayesian estimations of origination and extinction rates through time as inferred by PyRate using reversible jump Markov Chain Monte Carlo. Marginal estimates of origination rates (A) and extinction rates (C) through time shown as mean and 95% CI (shaded areas). Frequency of a sampled rate shift computed within small time bins for origination (B) and extinction rates (D), with horizontal dashed lines indicating log-Bayes factors of 2 (bottom) and 6 (top). Sampling frequencies higher than log-Bayes factors = 6 indicate strong statistical support for a rate shift. Panel (E) indicates posterior difference between origination and extinction rates through time

defined as net diversification rates through time. Panel (F) represents number of genera through time computed by summing up lifespans of all genera. Time-unit is millions of years (Myr). Colors of geological periods in chronostratigraphic scale following International Chronostratigraphic Chart (ICS, v2023/09).

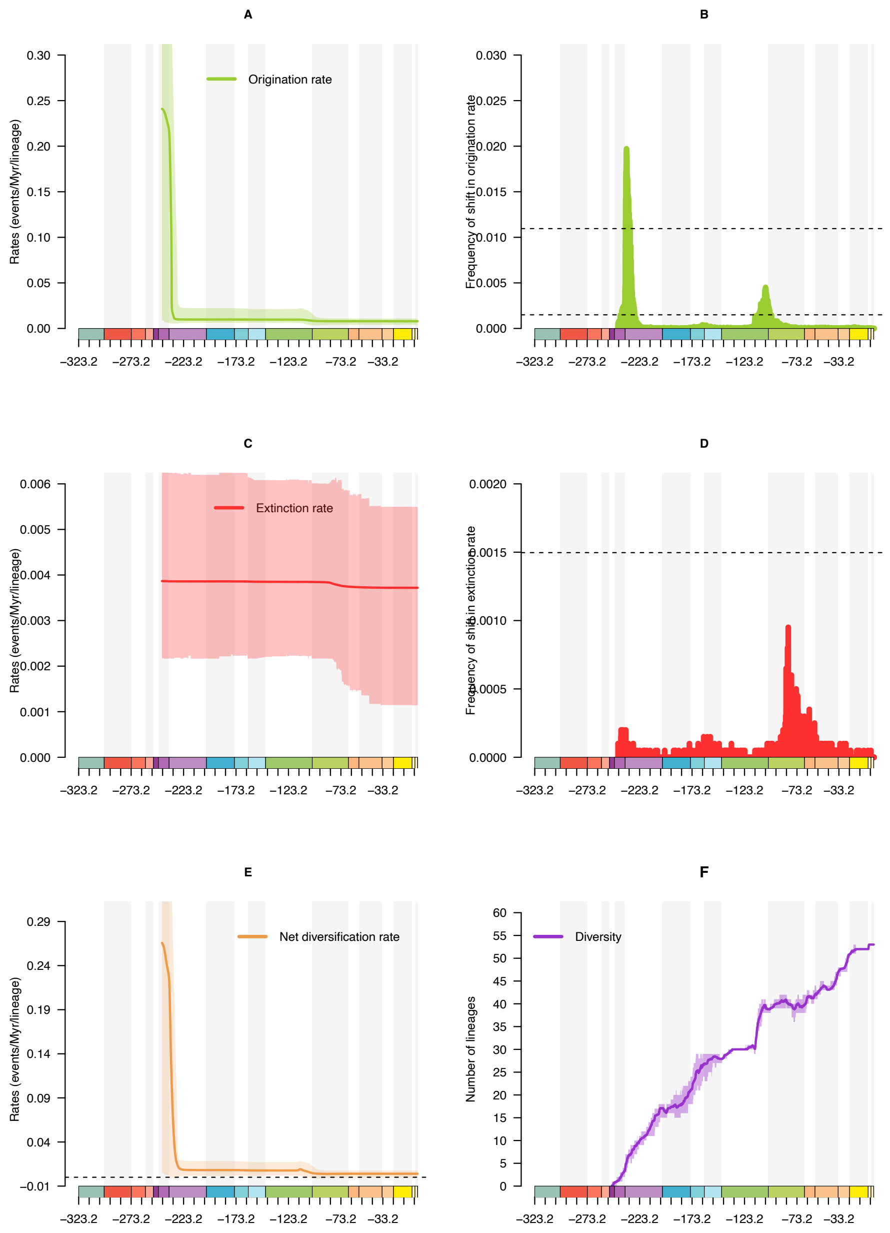

Supplementary Figure 12. Diversification and diversity dynamics of Heteroptera (family-level analysis). Bayesian estimations of origination and extinction rates through time as inferred by PyRate using reversible jump Markov Chain Monte Carlo. Marginal estimates of origination rates (A) and extinction rates (C) through time shown as mean and 95% CI (shaded areas). Frequency of a sampled rate shift computed within small time bins for origination (B) and extinction rates (D), with horizontal dashed lines indicating log-Bayes factors of 2 (bottom) and 6 (top). Sampling frequencies higher than log-Bayes factors = 6 indicate strong statistical support for a rate shift.

Panel (E) indicates posterior difference between origination and extinction rates through time defined as net diversification rates through time. Panel (F) represents number of genera through time computed by summing up lifespans of all genera. Time-unit is millions of years (Myr). Colors of geological periods in chronostratigraphic scale following International Chronostratigraphic Chart (ICS, v2023/09).

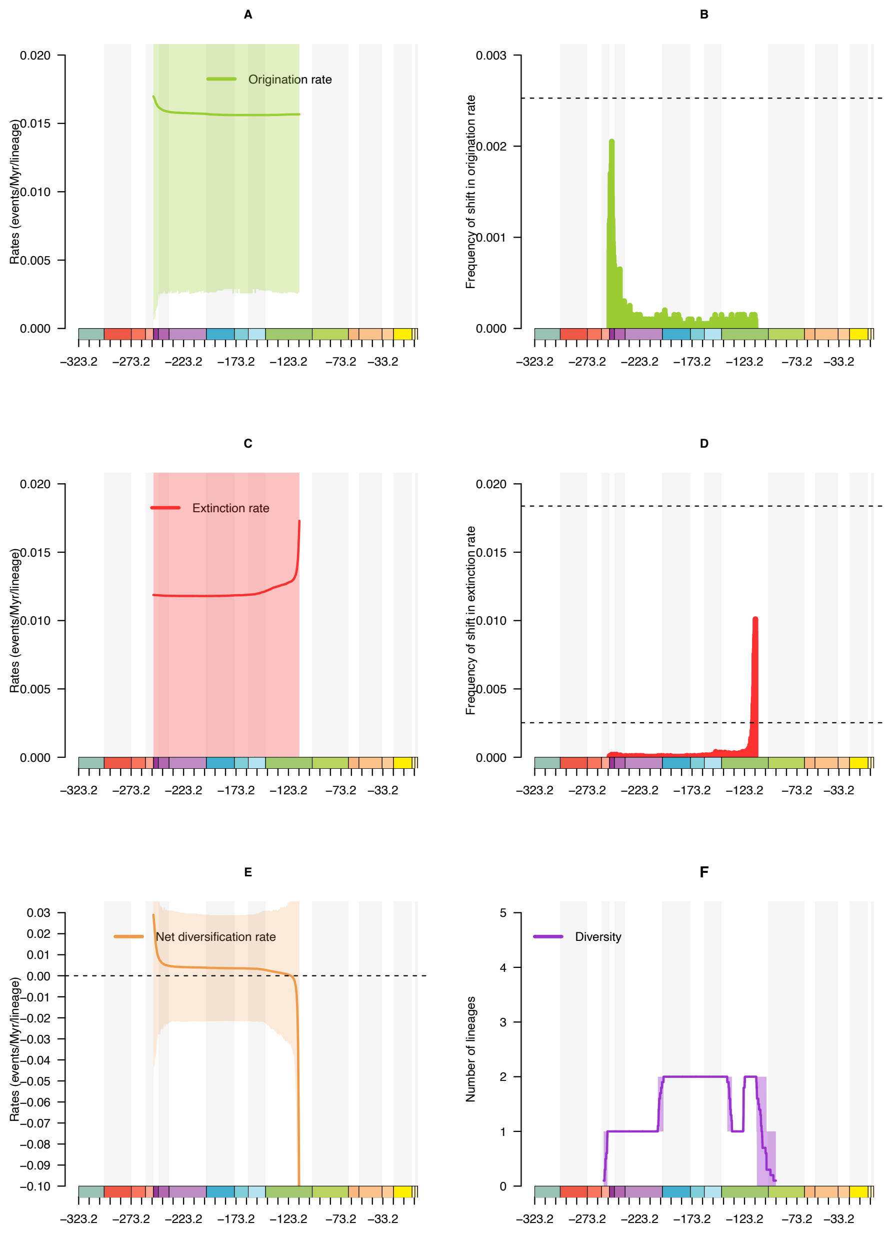

Supplementary Figure 13. Diversification and diversity dynamics of Coleorrhyncha (family-level analysis). Bayesian estimations of origination and extinction rates through time as inferred by PyRate using reversible jump Markov Chain Monte Carlo. Marginal estimates of origination rates (A) and extinction rates (C) through time shown as mean and 95% CI (shaded areas). Frequency of a sampled rate shift computed within small time bins for origination (B) and extinction rates (D), with horizontal dashed lines indicating log-Bayes factors of 2 (bottom) and 6 (top). Sampling frequencies higher than log-Bayes factors = 6 indicate strong statistical support for a rate shift. Panel (E) indicates posterior difference between origination and extinction rates through time

defined as net diversification rates through time. Panel (F) represents number of genera through time computed by summing up lifespans of all genera. Time-unit is millions of years (Myr). Colors of geological periods in chronostratigraphic scale following International Chronostratigraphic Chart (ICS, v2023/09).

Supplementary Table 1. Posterior parameter estimates for the MBD model applied to Hemiptera (genus-level analysis). Baseline origination and extinction rates ($\lambda 0$ and $\mu 0$) and correlation parameters ($G\lambda$ and $G\mu$). The drivers are numbered as follows: (0) diversity of all genera through time, (1) Angiosperms diversity through time, (2) Gymnosperms diversity through time, (3) Polypodiales ferns diversity through time, (4) Spore-plants diversity through time, (5) global temperature changes through time, and (6) non-Polypodiales ferns diversity through time. Shrinkage weights (ω), based on local and global shrinkage parameters, and global shrinkage (τ). The effect of a variable is assessed as significant when the shrinkage weight is superior to 0.5 and 95% HPD does not encompass 0.

parameters		median	95% HPD interval
**baseline rates**	λ0	0.0248	[0.0143, 0.0382]
	μ0	0.0587	[0.0295, 0.1047]
**Correlation parameters to origination**	Gλ0_0	-0.8463	[-1.2212, -0.4356]
	Gλ0_1	1.8569	[1.2542, 2.473]
	Gλ0_2	-0.0611	[-0.7188, 0.42]
	Gλ0_3	-0.8661	[-1.8813, 0.1005]
	Gλ0_4	6	[2.2507, 6.4775]
	Gλ0_5	8.3423E-4	[-0.0119, 0.0163]
	Gλ0_6	3.6192	[2.7599, 4.4622]
**Correlation parameters to extinction**	Gμ0_0	-0.1192	[-0.5424, 0.2117]
	Gμ0_1	0.2912	[-0.2312, 1.1249]
	Gμ0_2	-0.8183	[-1.5685, 0.0493]
	Gμ0_3	-0.9328	[-2.0084, 0.1267]
	Gμ0_4	2.1976	[-0.2328, 4.4943]
	Gμ0_5	-0.0095	[-0.035, 8.3487E-3]
	Gμ0_6	3.5145	[2.4005, 4.6946]
**Shrinkage weights (origination)**	ωλ0_0	0.5273	[0.1385, 0.9998]
	ωλ0_1	0.7876	[0.4, 0.9999]
	ωλ0_2	0.1019	[5.9204E-10, 0.8526]
	ωλ0_3	0.5507	[3.7827E-8, 0.9598]
	ωλ0_4	0.8771	[0.5478, 1]
	ωλ0_5	0.1515	[5.4178E-9, 0.8785]
	ωλ0_6	0.8085	[0.4521, 1]
**Shrinkage weights (extinction)**	ωμ0_0	0.1386	[2.9475E-8, 0.8678]
	ωμ0_1	0.2679	[8.6042E-8, 0.9224]
	ωμ0_2	0.3706	[1.3203E-7, 0.931]
	ωμ0_3	0.5595	[1.4861E-7, 0.9635]
	ωμ0_4	0.6946	[0.0766, 1]
	ωμ0_5	0.3262	[3.3844E-7, 0.9333]
	ωμ0_6	0.8014	[0.4287, 0.9999]
**Global shrinkage Hyperprior**	τ	1.0528	[0.3183, 2.2035]
	η	4.5126	[1.9897, 9.4118]

Supplementary Table 2. Posterior parameter estimates for the MBD model applied to Hemiptera (family-level analysis). Baseline origination and extinction rates ($\lambda0$ and $\mu0$) and correlation parameters ($G\lambda$ and $G\mu$). The drivers are numbered as follows: (0) diversity of all Hetm through time, (1) Angiosperms diversity through time, (2) Gymnosperms diversity through time, (3) Polypodiales ferns diversity through time, (4) Spore-plants diversity through time, (5) global temperature changes through time, and (6) non-Polypodiales ferns diversity through time. Shrinkage weights (ω), based on local and global shrinkage parameters, and global shrinkage (τ). The effect of a variable is assessed as significant when the shrinkage weight is superior to 0.5 and 95% HPD does not encompass 0.

parameters		median	95% HPD interval
**baseline rates**	λ0	0.0173	[5.0994E-3, 0.0403]
	μ0	0.0115	[6.9903E-4, 0.0404]
**Correlation parameters to origination**	Gλ0_0	-0.9277	[-2.4635, 0.1548]
	Gλ0_1	0.0142	[-0.8356, 1.1632]
	Gλ0_2	-0.0228	[-1.1044, 0.9378]
	Gλ0_3	-0.2444	[-2.0266, 0.9815]
	Gλ0_4	5.699	[2.073, 9.9552]
	Gλ0_5	-0.0014	[-0.0313, 0.0226]
	Gλ0_6	0.604	[-0.2886, 2.4894]
**Correlation parameters to extinction**	Gμ0_0	4.1767	[1.032, 6.5723]
	Gμ0_1	-3.5427	[-6.1551, -0.6212]
	Gμ0_2	-3.3941	[-5.5231, -1.1891]
	Gμ0_3	-2.7682	[-6.5729, 0.2834]
	Gμ0_4	6.7854	[-0.1187, 11.5365]
	Gμ0_5	2.7829E-3	[-0.0295, 0.0638]
	Gμ0_6	-1.0791	[-3.6788, 0.8903]
**Shrinkage weights (origination)**	ωλ0_0	0.6209	[0.0231, 0.9932]
	ωλ0_1	0.237	[8.7133E-7, 0.9314]
	ωλ0_2	0.1842	[4.8618E-7, 0.887]
	ωλ0_3	0.4007	[1.0524E-7, 0.9573]
	ωλ0_4	0.924	[0.5825, 0.9999]
	ωλ0_5	0.3056	[3.6226E-6, 0.9391]
	ωλ0_6	0.3364	[3.0932E-6, 0.9534]
**Shrinkage weights (extinction)**	ωμ0_0	0.9365	[0.622, 0.9997]
	ωμ0_1	0.9228	[0.5504, 1]
	ωμ0_2	0.8418	[0.3642, 1]
	ωμ0_3	0.8849	[0.041, 0.9996]
	ωμ0_4	0.9387	[0.403, 0.9999]
	ωμ0_5	0.4258	[5.075E-7, 0.9661]
	ωμ0_6	0.5007	[1.635E-6, 0.9623]
**Global shrinkage Hyperprior**	τ	1.4023	[0.4362, 3.3481]
	η	4.2914	[2.904, 6.0404]

Supplementary Table 3. Posterior parameter estimates for the MBD model applied to Auchenorrhyncha (genus-level analysis). Baseline origination and extinction rates ($\lambda 0$ and $\mu 0$) and correlation parameters ($G\lambda$ and $G\mu$). Drivers numbered as follows: (0) diversity of all Sternorrhyncha through time, (1) Angiosperms diversity through time, (2) Gymnosperms diversity through time, (3) Polypodiales ferns diversity through time, (4) Spore-plants diversity through time, (5) global temperature changes through time, and (6) non-Polypodiales ferns diversity through time. Shrinkage weights (ω), based on local and global shrinkage parameters, and global shrinkage (τ). The effect of a variable is assessed as significant when the shrinkage weight is superior to 0.5 and 95% HPD does not encompass 0.

parameters		median	95% HPD interval
**baseline rates**	λ0	0.0394	[0.015, 0.076]
	μ0	0.0514	[0.017, 0.1022]
**Correlation parameters to origination**	Gλ0_0	-1.2144	[-1.8569, -0.5452]
	Gλ0_1	1.8137	[0.8915, 2.8064]
	Gλ0_2	-1.7519	[-3.0536, -0.5236]
	Gλ0_3	-1.0039	[-2.4014, 0.2559]
	Gλ0_4	2.8966	[-0.2528, 6.1274]
	Gλ0_5	-0.0001	[-0.0245, 0.0227]
	Gλ0_6	4.8375	[3.2686, 6.3973]
**Correlation parameters to extinction**	Gμ0_0	-0.5178	[-1.1979, 0.1293]
	Gμ0_1	0.586	[-0.2022, 1.7346]
	Gμ0_2	-1.5582	[-2.8763, -0.1404]
	Gμ0_3	-0.5056	[-2.2432, 0.4565]
	Gμ0_4	0.6466	[-1.125, 4.0608]
	Gμ0_5	-0.002	[-0.0334, 0.0184]
	Gμ0_6	4.2322	[2.4682, 6.195]
**Shrinkage weights (origination)**	ωλ0_0	0.6784	[0.2332, 1]
	ωλ0_1	0.7905	[0.3635, 0.9999]
	ωλ0_2	0.6579	[0.1685, 0.9999]
	ωλ0_3	0.6132	[0.0282, 1]
	ωλ0_4	0.7877	[0.0818, 1]
	ωλ0_5	0.2314	[6.528E-8, 0.9192]
	ωλ0_6	0.8781	[0.5859, 0.9999]
**Shrinkage weights (extinction)**	ωμ0_0	0.4071	[8.0666E-9, 0.9429]
	ωμ0_1	0.4561	[6.4636E-7, 0.955]
	ωμ0_2	0.6144	[0.1154, 0.9999]
	ωμ0_3	0.4614	[7.6804E-7, 0.961]
	ωμ0_4	0.4605	[2.2476E-7, 0.9584]
	ωμ0_5	0.2726	[7.9164E-10, 0.9373]
	ωμ0_6	0.8527	[0.4988, 1]
**Global shrinkage Hyperprior**	τ	1.2565	[0.4019, 2.6118]
	η	4.8555	[2.2407, 8.8274]

Supplementary Table 4. Posterior parameter estimates for the MBD model applied to Auchenorrhyncha (family-level analysis). Baseline origination and extinction rates ($\lambda 0$ and $\mu 0$) and correlation parameters ($G\lambda$ and $G\mu$). Drivers numbered as follows: (0) diversity of all Auchenorrhyncha through time, (1) Angiosperms diversity through time, (2) Gymnosperms diversity through time, (3) Polypodiales ferns diversity through time, (4) Spore-plants diversity through time, (5) global temperature changes through time, and (6) non-Polypodiales ferns diversity through time. Shrinkage weights (ω), based on local and global shrinkage parameters, and global shrinkage (τ). The effect of a variable is assessed as significant when the shrinkage weight is superior to 0.5 and 95% HPD does not encompass 0.

parameters		median	95% HPD interval
**baseline rates**	λ0	0.05	[3.5875E-3, 0.1526]
	μ0	0.0192	[1.6837E-4, 0.0964]
**Correlation parameters to origination**	Gλ0_0	-2.5516	[-4.3806, 0.0542]
	Gλ0_1	0.0135	[-1.1629, 1.629]
	Gλ0_2	-0.0704	[-2.0167, 1.1591]
	Gλ0_3	0.0461	[-1.3053, 1.9862]
	Gλ0_4	0.9613	[-1.1783, 7.4988]
	Gλ0_5	-0.0001	[-0.0341, 0.0315]
	Gλ0_6	0.3977	[-0.8459, 3.0877]
**Correlation parameters to extinction**	Gμ0_0	0.3012	[-1.2184, 7.2583]
	Gμ0_1	-2.0102	[-9.386, 0.7847]
	Gμ0_2	-0.2309	[-7.2745, 1.5754]
	Gμ0_3	-0.4254	[-4.3932, 1.3429]
	Gμ0_4	-0.2621	[-8.9406, 2.5833]
	Gμ0_5	3.5263E-3	[-0.0303, 0.0685]
	Gμ0_6	-0.2212	[-3.6175, 1.3908]
**Shrinkage weights (origination)**	ωλ0_0	0.8404	[0.261, 1]
	ωλ0_1	0.1752	[6.3798E-9, 0.9066]
	ωλ0_2	0.148	[1.392E-9, 0.8831]
	ωλ0_3	0.2058	[5.2037E-8, 0.9299]
	ωλ0_4	0.4662	[3.2918E-9, 0.9794]
	ωλ0_5	0.1696	[3.8227E-9, 0.8995]
	ωλ0_6	0.2187	[2.106E-10, 0.9248]
**Shrinkage weights (extinction)**	ωμ0_0	0.374	[5.2257E-8, 0.9867]
	ωμ0_1	0.7875	[0.0122, 1]
	ωμ0_2	0.2738	[9.3778E-9, 0.9737]
	ωμ0_3	0.4739	[4.7805E-8, 0.9755]
	ωμ0_4	0.3012	[2.0887E-9, 0.9802]
	ωμ0_5	0.2583	[2.0693E-9, 0.9477]
	ωμ0_6	0.2108	[1.2627E-9, 0.9267]
**Global shrinkage Hyperprior**	τ	0.6978	[0.0542, 2.2451]
	η	5.4069	[2.4662, 10.4294]

Supplementary Table 5. Posterior parameter estimates for the MBD model applied to Sternorrhyncha (genus-level analysis). Baseline origination and extinction rates ($\lambda 0$ and $\mu 0$) and correlation parameters ($G\lambda$ and $G\mu$). Drivers numbered as follows: (0) diversity of all family through time, (1) Sternorrhyncha diversity through time, (2) Gymnosperms diversity through time, (3) Polypodiales ferns diversity through time, (4) Spore-plants diversity through time, (5) global temperature changes through time, and (6) non-Polypodiales ferns diversity through time. Shrinkage weights (ω), based on local and global shrinkage parameters, and global shrinkage (τ). The effect of a variable is assessed as significant when the shrinkage weight is superior to 0.5 and 95% HPD does not encompass 0.

parameters		median	95% HPD interval
**baseline rates**	λ0	0.0219	[1.0647E-3, 0.057]
	μ0	0.0323	[7.6939E-3, 0.0882]
**Correlation parameters to origination**	Gλ0_0	-0.1131	[-0.9769, 0.4318]
	Gλ0_1	3.8448	[1.2412, 5.4888]
	Gλ0_2	0.6117	[-0.6633, 2.5266]
	Gλ0_3	-4.0999	[-6.637, 0.9224]
	Gλ0_4	7.6882	[-0.2359, 15.4256]
	Gλ0_5	-0.0001	[-0.0336, 0.0336]
	Gλ0_6	2.9615	[-0.0725, 5.1517]
**Correlation parameters to extinction**	Gμ0_0	0.0835	[-0.5396, 0.9889]
	Gμ0_1	4.7197E-3	[-1.0939, 1.0487]
	Gμ0_2	-0.806	[-3.662, 0.8016]
	Gμ0_3	-0.0984	[-1.8813, 1.0755]
	Gμ0_4	11.2745	[5.4171, 17.3977]
	Gμ0_5	-0.017	[-0.0637, 0.0115]
	Gμ0_6	2.2093	[-0.258, 5.9317]
**Shrinkage weights (origination)**	ωλ0_0	0.2376	[4.1286E-9, 0.9219]
	ωλ0_1	0.9227	[0.6117, 1]
	ωλ0_2	0.4002	[2.282E-8, 0.9494]
	ωλ0_3	0.9306	[0.3918, 1]
	ωλ0_4	0.953	[0.5194, 1]
	ωλ0_5	0.3134	[3.7749E-8, 0.9427]
	ωλ0_6	0.7653	[0.1172, 1]
**Shrinkage weights (extinction)**	ωμ0_0	0.2073	[1.6042E-10, 0.9223]
	ωμ0_1	0.2663	[2.9468E-8, 0.9369]
	ωμ0_2	0.4853	[7.2419E-9, 0.9679]
	ωμ0_3	0.3412	[3.8192E-9, 0.9515]
	ωμ0_4	0.9758	[0.868, 1]
	ωμ0_5	0.5381	[5.5019E-8, 0.9715]
	ωμ0_6	0.7024	[0.043, 1]
**Global shrinkage Hyperprior**	τ	1.3282	[0.3028, 3.1285]
	η	6.3958	[2.6476, 13.9172]

Supplementary Table 6. Posterior parameter estimates for the MBD model applied to Sternorrhyncha (family-level analysis). Baseline origination and extinction rates ($\lambda 0$ and $\mu 0$) and correlation parameters ($G\lambda$ and $G\mu$). Drivers numbered as follows: (0) diversity of all Sternorrhyncha through time, (1) Angiosperms diversity through time, (2) Gymnosperms diversity through time, (3) Polypodiales ferns diversity through time, (4) Spore-plants diversity through time, (5) global temperature changes through time, and (6) non-Polypodiales ferns diversity through time. Shrinkage weights (ω), based on local and global shrinkage parameters, and global shrinkage (τ). The effect of a variable is assessed as significant when the shrinkage weight is superior to 0.5 and 95% HPD does not encompass 0.

parameters		median	95% HPD interval
**baseline rates**	λ0	0.0351	[4.2664E-3, 0.1174]
	μ0	0.0654	[2.0127E-3, 0.4029]
**Correlation parameters to origination**	Gλ0_0	-0.13	[-1.4828, 0.9325]
	Gλ0_1	-0.0085	[-1.5744, 1.7002]
	Gλ0_2	-0.4246	[-2.8865, 0.9289]
	Gλ0_3	-1.5324	[-4.448, 0.5106]
	Gλ0_4	0.4231	[-2.8444, 6.5768]
	Gλ0_5	-0.0014	[-0.0479, 0.0322]
	Gλ0_6	1.5122	[-0.6214, 5.0174]
**Correlation parameters to extinction**	Gμ0_0	3.7487	[0.8684, 6.7372]
	Gμ0_1	-1.2532	[-4.5958, 0.6461]
	Gμ0_2	-2.7432	[-5.4469, 0.181]
	Gμ0_3	-6.0795	[-11.504, 0.3058]
	Gμ0_4	0.2875	[-3.3196, 9.5181]
	Gμ0_5	-0.0048	[-0.0787, 0.035]
	Gμ0_6	-0.3588	[-5.18, 2.137]
**Shrinkage weights (origination)**	ωλ0_0	0.2947	[1.4303E-8, 0.9316]
	ωλ0_1	0.3107	[9.1505E-13, 0.9437]
	ωλ0_2	0.3323	[4.6297E-9, 0.9533]
	ωλ0_3	0.7348	[0.022, 1]
	ωλ0_4	0.5016	[1.2493E-7, 0.9765]
	ωλ0_5	0.3039	[2.5218E-9, 0.9486]
	ωλ0_6	0.554	[1.782E-6, 0.9724]
**Shrinkage weights (extinction)**	ωμ0_0	0.9234	[0.5219, 1]
	ωμ0_1	0.7025	[1.8175E-8, 0.985]
	ωμ0_2	0.7678	[0.0977, 0.9998]
	ωμ0_3	0.9632	[0.2654, 1]
	ωμ0_4	0.4876	[4.2583E-9, 0.9834]
	ωμ0_5	0.414	[1.0028E-8, 0.9654]
	ωμ0_6	0.4003	[2.2706E-9, 0.9629]
**Global shrinkage Hyperprior**	τ	1.1447	[0.1925, 2.9244]
	η	5.5859	[2.2532, 14.499]

Supplementary Table 7. Posterior parameter estimates for the MBD model applied to Heteroptera (genus-level analysis). Baseline origination and extinction rates ($\lambda 0$ and $\mu 0$) and correlation parameters ($G\lambda$ and $G\mu$). Drivers numbered as follows: (0) diversity of all Heteroptera through time, (1) Angiosperms diversity through time, (2) Gymnosperms diversity through time, (3) Polypodiales ferns diversity through time, (4) Spore-plants diversity through time, (5) global temperature changes through time, and (6) non-Polypodiales ferns diversity through time. Shrinkage weights (ω), based on local and global shrinkage parameters, and global shrinkage (τ). The effect of a variable is assessed as significant when the shrinkage weight is superior to 0.5 and 95% HPD does not encompass 0.

parameters		median	95% HPD interval
**baseline rates**	λ0	0.2428	[0.0832, 0.4697]
	μ0	0.5534	[0.1797, 1.1651]
**Correlation parameters to origination**	Gλ0_0	-1.4056	[-1.976, -0.8814]
	Gλ0_1	0.3128	[-0.2626, 1.2112]
	Gλ0_2	0.4531	[-0.3073, 1.6052]
	Gλ0_3	-1.4088	[-2.764, 0.0472]
	Gλ0_4	3.6307	[-0.7869, 9.2077]
	Gλ0_5	-0.0013	[-0.0265, 0.0167]
	Gλ0_6	-0.0731	[-1.4783, 0.937]
**Correlation parameters to extinction**	Gμ0_0	-0.7088	[-1.3316, 0.0104]
	Gμ0_1	-0.7717	[-1.9617, 0.209]
	Gμ0_2	0.0142	[-1.1467, 1.0677]
	Gμ0_3	-1.8839	[-3.5628, 0.069]
	Gμ0_4	0.0468	[-3.3716, 4.0905]
	Gμ0_5	-0.0296	[-0.0646, 2.534E-3]
	Gμ0_6	0.3749	[-0.8069, 2.2076]
**Shrinkage weights (origination)**	ωλ0_0	0.6779	[0.2688, 0.9998]
	ωλ0_1	0.2466	[6.9641E-8, 0.9039]
	ωλ0_2	0.2107	[1.378E-9, 0.8965]
	ωλ0_3	0.6686	[0.0833, 1]
	ωλ0_4	0.8205	[0.0191, 1]
	ωλ0_5	0.1641	[5.6943E-9, 0.8721]
	ωλ0_6	0.1332	[2.4095E-8, 0.8417]
**Shrinkage weights (extinction)**	ωμ0_0	0.4229	[5.8017E-9, 0.9239]
	ωμ0_1	0.4618	[1.398E-9, 0.9435]
	ωμ0_2	0.1331	[4.6322E-9, 0.8608]
	ωμ0_3	0.7555	[0.0764, 1]
	ωμ0_4	0.2856	[1.0698E-7, 0.9496]
	ωμ0_5	0.6146	[3.9796E-8, 0.9692]
	ωμ0_6	0.2194	[3.6458E-8, 0.8969]
**Global shrinkage Hyperprior**	τ	0.8251	[0.2192, 1.847]
	η	3.9164	[1.9548, 6.3431]

Supplementary Table 8. Posterior parameter estimates for the MBD model applied to Heteroptera (family-level analysis). Baseline origination and extinction rates ($\lambda 0$ and $\mu 0$) and correlation parameters ($G\lambda$ and $G\mu$). Drivers numbered as follows: (0) diversity of all Heteroptera through time, (1) Angiosperms diversity through time, (2) Gymnosperms diversity through time, (3) Polypodiales ferns diversity through time, (4) Spore-plants diversity through time, (5) global temperature changes through time, and (6) non-Polypodiales ferns diversity through time. Shrinkage weights (ω), based on local and global shrinkage parameters, and global shrinkage (τ). The effect of a variable is assessed as significant when the shrinkage weight is superior to 0.5 and 95% HPD does not encompass 0.

parameters		median	95% HPD interval
**baseline rates**	λ0	0.1224	[0.0219, 0.3644]
	μ0	0.0279	[8.1425E-4, 0.1957]
**Correlation parameters to origination**	Gλ0_0	-4.345	[-6.7658, -2.2967]
	Gλ0_1	0.2353	[-0.7949, 2.0991]
	Gλ0_2	-0.1784	[-2.1297, 1.0465]
	Gλ0_3	0.2126	[-1.041, 3.0713]
	Gλ0_4	0.0453	[-3.6317, 4.4165]
	Gλ0_5	-0.0017	[-0.0422, 0.0234]
	Gλ0_6	0.1528	[-1.2814, 3.2153]
**Correlation parameters to extinction**	Gμ0_0	-0.0574	[-2.7167, 1.9294]
	Gμ0_1	-0.5544	[-4.4507, 0.9671]
	Gμ0_2	-1.1516	[-5.1738, 0.7937]
	Gμ0_3	-1.7854	[-6.3401, 0.8531]
	Gμ0_4	-0.0776	[-7.8567, 4.2321]
	Gμ0_5	-0.0014	[-0.0617, 0.0346]
	Gμ0_6	-0.1772	[-4.5307, 1.8461]
**Shrinkage weights (origination)**	ωλ0_0	0.9395	[0.7226, 1]
	ωλ0_1	0.2585	[1.2856E-8, 0.9328]
	ωλ0_2	0.1792	[1.6351E-8, 0.8867]
	ωλ0_3	0.3204	[6.9953E-9, 0.9552]
	ωλ0_4	0.2649	[2.617E-8, 0.953]
	ωλ0_5	0.1894	[2.1184E-9, 0.9065]
	ωλ0_6	0.1817	[4.389E-9, 0.9161]
**Shrinkage weights (extinction)**	ωμ0_0	0.2876	[2.3848E-11, 0.9483]
	ωμ0_1	0.4844	[7.3752E-11, 0.9763]
	ωμ0_2	0.4899	[2.5025E-9, 0.9702]
	ωμ0_3	0.7573	[1.2629E-7, 0.9895]
	ωμ0_4	0.3237	[5.9919E-9, 0.9739]
	ωμ0_5	0.2418	[3.885E-8, 0.9347]
	ωμ0_6	0.2392	[3.121E-9, 0.932]
**Global shrinkage Hyperprior**	τ	0.7574	[0.0916, 2.0255]
	η	5.1862	[2.4659, 9.6784]
