# Supplementary material for: Diversification and extinction of Hemiptera in deep time
Source: Commun Biol. 2025 Mar 3;8:352. doi: 10.1038/s42003-025-07773-x (PMC11876582; doi:10.1038/s42003-025-07773-x)
Supplement: Supplementary file 2 — Reporting Summary [file 42003_2025_7773_MOESM2_ESM.pdf]

nature portfolio

Corresponding author(s): Mathieu Boderau

Last updated by author(s): Feb 11, 2025

Reporting Summary

Nature Portfolio wishes to improve the reproducibility of the work that we publish. This form provides structure for consistency and transparency in reporting. For further information on Nature Portfolio policies, see our Editorial Policies and the Editorial Policy Checklist.

Statistics

For all statistical analyses, confirm that the following items are present in the figure legend, table legend, main text, or Methods section.

n/a	Confirmed	
☐	☒	The exact sample size (*n*) for each experimental group/condition, given as a discrete number and unit of measurement
☒	☐	A statement on whether measurements were taken from distinct samples or whether the same sample was measured repeatedly
☒	☐	The statistical test(s) used AND whether they are one- or two-sided<br>*Only common tests should be described solely by name; describe more complex techniques in the Methods section.*
☐	☒	A description of all covariates tested
☒	☐	A description of any assumptions or corrections, such as tests of normality and adjustment for multiple comparisons
☐	☒	A full description of the statistical parameters including central tendency (e.g. means) or other basic estimates (e.g. regression coefficient) AND variation (e.g. standard deviation) or associated estimates of uncertainty (e.g. confidence intervals)
☒	☐	For null hypothesis testing, the test statistic (e.g. *F*, *t*, *r*) with confidence intervals, effect sizes, degrees of freedom and *P* value noted<br>*Give P values as exact values whenever suitable.*
☐	☒	For Bayesian analysis, information on the choice of priors and Markov chain Monte Carlo settings
☒	☐	For hierarchical and complex designs, identification of the appropriate level for tests and full reporting of outcomes
☒	☐	Estimates of effect sizes (e.g. Cohen's *d*, Pearson's *r*), indicating how they were calculated

Our web collection on statistics for biologists contains articles on many of the points above.

Software and code

Policy information about availability of computer code

Data collection	We collected the fossil data from the Paleobiology Database (https://paleobiodb.org/), extracted all occurences for 'Hemiptera', cleaned this first dataset based on our expertise and enhanced it with an intensive mining of the bibliography.
Data analysis	We used PyRate v.3.1.3 (https://github.com/dsilvestro/PyRate) to conduct all analyses. PyRate is a Bayesian model to estimate origination, extinction and preservation rates from fossil occurrence data.

For manuscripts utilizing custom algorithms or software that are central to the research but not yet described in published literature, software must be made available to editors and reviewers. We strongly encourage code deposition in a community repository (e.g. GitHub). See the Nature Portfolio guidelines for submitting code & software for further information.

Data

Policy information about availability of data

All manuscripts must include a data availability statement. This statement should provide the following information, where applicable:

- Accession codes, unique identifiers, or web links for publicly available datasets
- A description of any restrictions on data availability
- For clinical datasets or third party data, please ensure that the statement adheres to our policy

Datasets are available as Supplementary Files and can be found on the publisher's website.

Human research participants

Policy information about studies involving human research participants and Sex and Gender in Research.

Reporting on sex and gender	N/A
Population characteristics	N/A
Recruitment	N/A
Ethics oversight	N/A

Note that full information on the approval of the study protocol must also be provided in the manuscript.

Field-specific reporting

Please select the one below that is the best fit for your research. If you are not sure, read the appropriate sections before making your selection.

☐ Life sciences ☐ Behavioural & social sciences ☒ Ecological, evolutionary & environmental sciences

For a reference copy of the document with all sections, see nature.com/documents/nr-reporting-summary-flat.pdf

Ecological, evolutionary & environmental sciences study design

All studies must disclose on these points even when the disclosure is negative.

Study description	We depicted the temporal dynamics (origination and extinction) and the diversification patterns through deep time of Hemiptera (cicadas, true hoppers, true bugs, aphids, etc...), the fifth most diversified insect lineage. We investigated the impact of abiotic and biotic factors driving their evolutionnary history. Our study is based on a Bayesian fossil-based approach explicitly modelling preservation rates as well as times of origination and extinction of each genus.
Research sample	All occurences of Hemiptera fossils.
Sampling strategy	Sample size was determined by availability of fossil data for Hemiptera. We used all occurences available in our study. The final size of our dataset fits with the sample size of most of published fossil-based diversification studies.
Data collection	We initially extracted the fossils occurrences of Hemiptera from The Palaeobiology Database (PBDB, https://paleobiodb.org/; downloaded in March 2022). All synonyms, outdated combinations, nomina dubia, and other erroneous and doubtful records, were corrected. Numerous occurrences of fossils attributed to species were revised to genus- or family- level occurrences. We surveyed the bibliography and visited institutional collections to enhance our dataset. The final dataset encompasses 11,842 fossil occurrences for 244 families and 1,794 genera ranging from Pennsylvanian (Carboniferous) to Holocene.
Timing and spatial scale	Timing scale of this study correspond to the first period recording Hemiptera fossils (Carboniferous) to Holocene (present). The study's spatial scale is a global scale (all occurences of Hemiptera without any geographical assignation).
Data exclusions	We excluded taxa that are nomina dubia, which cannot be attributed to a genus or family of Hemiptera.
Reproducibility	Our data are not experimental, subsequently experiments were not replicated.
Randomization	We didn't carry out any experiments, so there was no group allocation. We used all insect taxa for which fossil occurrence data were available.
Blinding	Bliding is not relevant to our study, because we used all available data.

Did the study involve field work? ☐ Yes ☒ No

Reporting for specific materials, systems and methods

We require information from authors about some types of materials, experimental systems and methods used in many studies. Here, indicate whether each material, system or method listed is relevant to your study. If you are not sure if a list item applies to your research, read the appropriate section before selecting a response.

Materials & experimental systems

n/a	Involved in the study	
☒	☐	Antibodies
☒	☐	Eukaryotic cell lines
☐	☒	Palaeontology and archaeology
☒	☐	Animals and other organisms
☒	☐	Clinical data
☒	☐	Dual use research of concern

Methods

n/a	Involved in the study	
☒	☐	ChIP-seq
☒	☐	Flow cytometry
☒	☐	MRI-based neuroimaging

Palaeontology and Archaeology

Specimen provenance	No new specimens are provided in this study. All datasets were initally compiled from the Paleobiology Database (https://paleobiodb.org/).
Specimen deposition	All specimens mentionned in our dataset are all already deposited in public institutions (Museums) and/or referenced in the Paleobiology Database (https://paleobiodb.org/).
Dating methods	We do not provided any new dates. Ages of fossil occurrences correspond to the age of stratigraphic horizon from which the fossil is originated, standardized at stage-level precision.

☒ Tick this box to confirm that the raw and calibrated dates are available in the paper or in Supplementary Information.

Ethics oversight	N/A

Note that full information on the approval of the study protocol must also be provided in the manuscript.
